# Emergence of a mupirocin-resistant, methicillin-susceptible *Staphylococcus aureus* clone associated with skin and soft tissue infections in Greece

**DOI:** 10.1186/s12866-021-02272-5

**Published:** 2021-07-03

**Authors:** Nikolaos Giormezis, Anastassios Doudoulakakis, Katerina Tsilipounidaki, Maria Militsopoulou, George Kalogeras, Vasiliki Stamouli, Fevronia Kolonitsiou, Efthimia Petinaki, Evangelia Lebessi, Iris Spiliopoulou

**Affiliations:** 1 Department of Microbiology, School of Medicine, University of Patras, 26504 Patras, Greece; 2 National Reference Laboratory for Staphylococci, University of Patras, Patras, Greece; 3 Department of Microbiology P. & A, Kyriakou Children’s Hospital, Athens, Greece; 4 Department of Microbiology, School of Medicine, University of Thessaly, Larissa, Greece; 5 Department of Microbiology, University General Hospital of Patras, Patras, Greece

**Keywords:** MSSA, SSTIs, Mupirocin-resistant, ST121, Exfoliative toxins, Greece

## Abstract

**Background:**

*Staphylococcus aureus* causes various infections, including skin and soft tissue infections (SSTIs). In this study, methicillin-susceptible *S. aureu*s (MSSA) from SSTIs among patients in three tertiary-care hospitals in Greece were studied in terms of antimicrobial resistance, clonal distribution, toxin and adhesin genes carriage.

**Results:**

During a five-year period (2014–2018), 6145 *S. aureus* were recovered from 13,244 patients with SSTIs and tested for antimicrobial susceptibility. MSSA were 4806 (78.21 %) including 1484 isolates with mupirocin minimum inhibitory concentration (MIC) > 64 mg/L (30.88 %). Two hundred and sixty representative mupirocin-resistant MSSA were analyzed for genes encoding Panton-Valentine leukocidin (PVL, *lukS*/*lukF-*PV), exfoliative toxins (*eta*, *etb*), adhesin FnbA (*fnbA*) and resistance genes *mupA* (high-level resistance to mupirocin), *fusB* (fusidic acid), aminoglycosides’ modifying enzymes, *ermA, ermC* and *msrA* (macrolides/lincosamides) by PCRs. Strains were classified into clones by PFGE and MLST.

All mupirocin-resistant MSSA were penicillin-resistant; 92.7 % expressed resistance to fusidic acid and 88.9 % to tobramycin. All 260 molecularly analyzed isolates were *mupA*-positive; all fusidic acid-resistant (241/260) carried *fusB* whereas, the tobramycin-resistant ones (230), *ant(4′)-Ia*. The majority carried *eta* (93.85 %), *etb* (98.08 %) and *fnbA* (88.85 %). PFGE typing revealed a mostly unvarying population; 260 MSSA were grouped into three types. One major *eta*/*etb*-positive clone comprising of 258/260 strains (99.2 %), PFGE type 1, was classified as ST121, including nine strains co-carrying PVL. Another PVL-positive strain was identified as ST1, and one toxins-negative as ST21.

**Conclusions:**

A mupirocin-resistant MSSA clone, ST121, carrying resistance, exfoliative toxins and adhesin genes, was spread and predominated in SSTIs from patients in Greece during the five-year studied period.

**Supplementary Information:**

The online version contains supplementary material available at 10.1186/s12866-021-02272-5.

## Background

*Staphylococcus aureus* is an extremely common bacterium in human population with around 20–30 % of people being carriers, more frequently in the anterior nasal cavity, but also the skin, the pharynx and the gastrointestinal tract [1]. Despite being part of the human flora, staphylococci can cause a wide range of infections, including skin and soft tissue infections (SSTIs), pneumonia and bacteraemia [2].

Several factors contribute to the establishment of staphylococcal infections. The initial attachment of the microorganism is promoted by adhesins grouped into the single family named Microbial Surface Components Recognizing Adhesive Matrix Molecules (MSCRAMMs) [3]. The *S. aureus* fibronectin-binding protein A (FnbA), encoded by *fnbA* gene, possesses multiple regions capable of conferring adherence to both soluble and immobilized forms of fibronectin [4]. This confers *S. aureus* the ability to invade endothelial cells *in vivo* and *in vitro*. Moreover, FnbA promotes bacterial attachment to fibrinogen as well as, adherence and aggregation of activated platelets [5]. Several studies have demonstrated the prevalence of FnbA against the homologous protein FnbB [6, 7].

Another important factor in the pathogenesis of staphylococcal infections is the production of exotoxins. Panton-Valentine leukocidin (PVL) is a cytotoxin produced by *S. aureus* that causes leukocyte destruction and tissue necrosis. The toxin is detected in large percentages among isolates that cause necrotic skin lesions and severe necrotizing pneumonia. Clinical and epidemiological studies suggest the involvement of PVL as an important factor contributing to the epidemic spread and virulence of community-acquired MRSA, however, this is still intensely debated [8]. The exfoliative toxins of *S. aureus* are responsible for the staphylococcal scalded skin syndrome (SSSS), a blistering skin disorder particularly affecting infants and young children, as well as adults with underlying disease. The target for the toxins has been identified as desmoglein-1, a desmosomal glycoprotein which plays an important role in maintaining cell-to-cell adhesion in the superficial epidermis. Exfoliative toxins A and B are the most frequently implicated in human skin damage [9].

Staphylococci express resistance to many antimicrobial agents, an increasing problem worldwide, especially among nosocomial pathogens. *S. aureus* is a leading cause of bacteremia and infective endocarditis as well as osteoarticular, skin and soft tissue, pleuropulmonary, and device-related infections [10]. From the first report on methicillin resistance in 1961 till today, methicillin-resistant staphylococci (MRSA) represent a major health problem worldwide [11]. However, methicillin-susceptible *S. aureus* (MSSA) are also often the cause of infections.

Mupirocin is an antibiotic from monocarboxylic acid class used as antibacterial agent against *S. aureus* and can be obtained as a mixture of four pseudomonic acids by *Pseudomonas fluorescens* biosynthesis. It is used to control the MRSA outbreaks, SSTIs and nasal decolonization [12]. Due to its wide use without prescription, the microorganism’s resistance to mupirocin increased from 1 to 81 %, thus compromising its place as therapeutic and decolonization agent [13]. In a previous study performed in a single pediatric hospital in Athens, Greece, a mupirocin-resistant ST121 clone among MSSA causing SSTIs was identified. This clone has emerged in 2013 and was detected to an increasing incidence till 2016 in this setting [14].

The observation that mupirocin-resistant MSSA were isolated with an increasing incidence since 2014 in two other tertiary care hospitals in Greece, led to the present study; MSSA with mupirocin minimum inhibitory concentration (MIC) > 1 mg/L recovered from SSTIs among patients admitted in three tertiary care hospitals in different areas of Greece (Athens, Southwestern and Central Greece) were studied in terms of antimicrobial resistance patterns, clonal distribution, toxins and adhesins gene carriage.

## Results

**Fig. 1:**
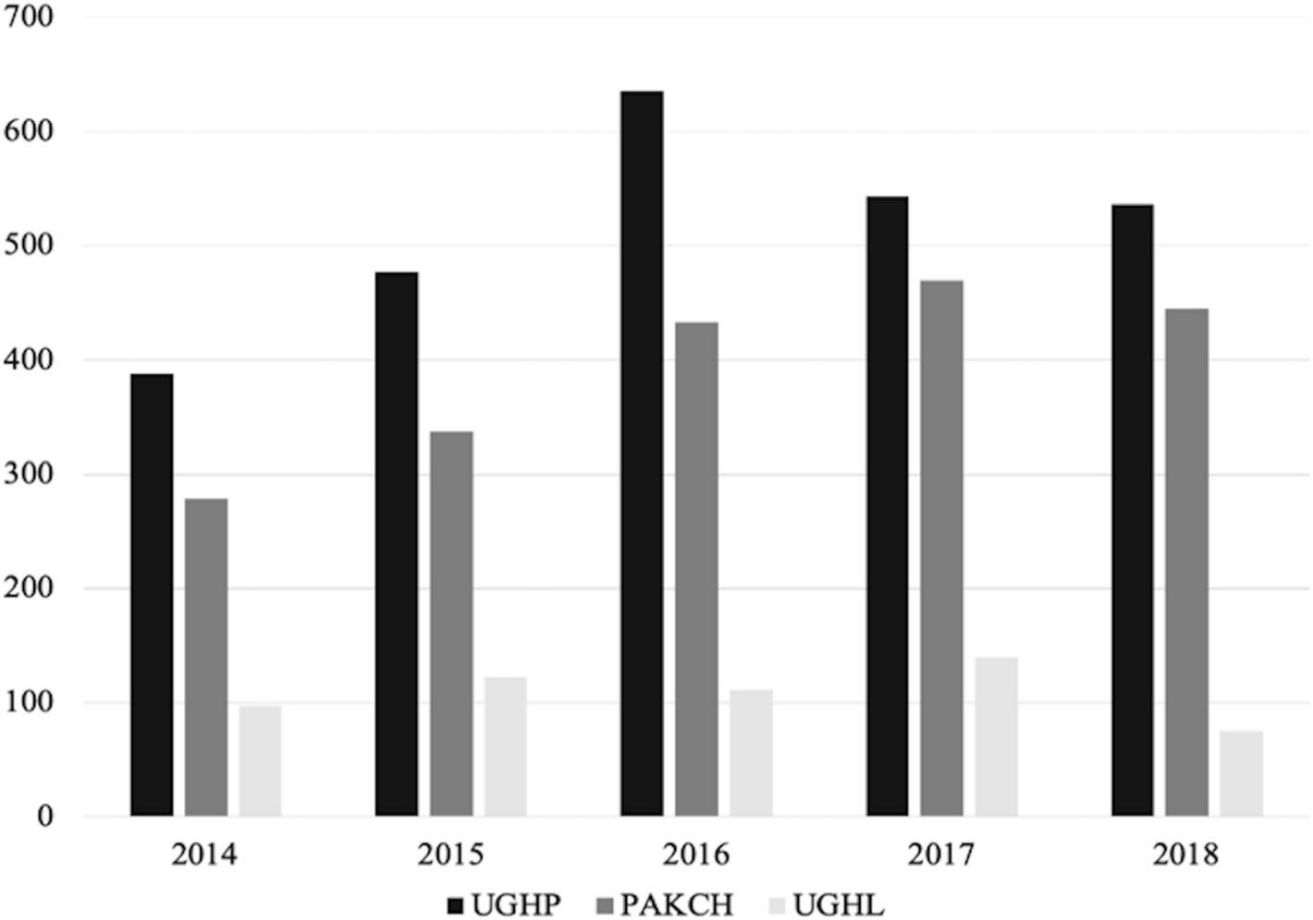
Number of *S. aureus* isolates recovered from patients with SSTIs, during the studied period (2014–2018). University General Hospital of Patras: UGHP; P. & A. Kyriakou Children’s Hospital: PAKCH; University General Hospital of Larissa: UGHL)

**Fig. 2:**
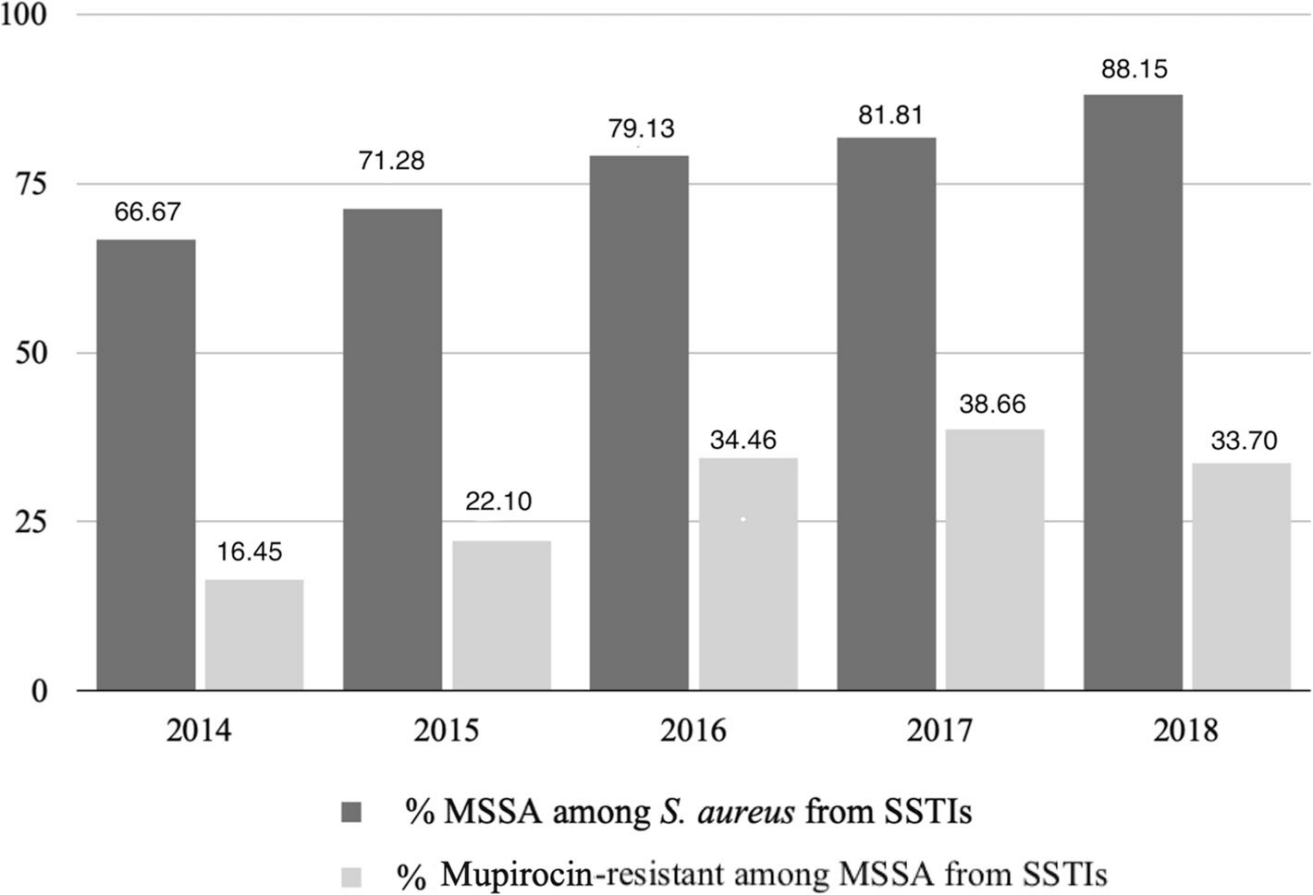
Annual distribution of MSSA and mupirocin-resistant MSSA recovered from SSTIs

From a total of 13,244 SSTIs recorded from 2014 to 2018, 6145 *S. aureu*s were identified (46.40 %), recovered from different patients. Of them, 4806 isolates (78.21 %) were MSSA. All cefoxitin-susceptible isolates were also oxacillin-susceptible. An increase in the isolation rate of *S. aureus* from patients with SSTIs was detected from 2014 to 2016, followed by a decrease in the next two years (Table 1; Fig. 1). An increase of MSSA among *S. aureus* causing SSTIs was also observed during the five-year period, from 66.67 % to 2014 to 88.15 % in 2018 (Fig. 2).

**Table 1:** Characteristics of recorded isolates in each participating hospital

	2014			2015			2016			2017			2018			TOTAL
	UGHP	PAKCH	UGHL	UGHP	PAKCH	UGHL	UGHP	PAKCH	UGHL	UGHP	PAKCH	UGHL	UGHP	PAKCH	UGHL	
	Number (%)	Number (%)	Number (%)	Number (%)	Number (%)	Number (%)	Number (%)	Number (%)	Number (%)	Number (%)	Number (%)	Number (%)	Number (%)	Number (%)	Number (%)	Number (%)
SSTIs	1111	335	697	1112	384	789	1443	509	986	1527	543	905	1505	511	887	13244
SA	388 (34.9)	279 (83.3)	254 (36.4)	477 (42.9)	337 (87.8)	335 (42.5)	635 (44.0)	433 (85.1)	384 (38.9)	543 (35.6)	470 (86.6)	378 (41.8)	536 (35.6)	445 (87.1)	251 (28.3)	6145 (46.4)
MSSA	246 (63.4)	211 (75.6)	157 (61.8)	347 (72.7)	274 (81.3)	198 (59.1)	527 (83)	379 (87.5)	243 (63.3)	468 (86.2)	430 (91.5)	240 (63.5)	519 (96.8)	390 (87.6)	177 (70.5)	4806 (78.2)
MUP-R	15 (6.1)	74 (35.1)	12 (7.6)	51 (14.7)	107 (39.1)	23 (11.6)	128 (24.3)	230 (60.7)	38 (15.6)	89 (19)	316 (73.5)	35 (14.6)	92 (17.7)	245 (62.8)	29 (16.4)	1484 (30.9)
MA	5 (33.3)	10 (13.5)	5 (41.7)	18 (35.3)	10 (9.3)	8 (34.8)	38 (29.7)	25 (10.9)	15 (39.5)	26 (29.2)	36 (11.4)	11 (31.4)	23 (25.0)	22 (9.0)	8 (27.6)	260 (17.5)

*SA S. aureus* from recorded SSTIs, *MSSA* Methicillin-susceptible isolates among *S. aureus*, *MUP-R* mupirocin-resistant strains among MSSA, *MA* molecularly analyzed strains

**Fig. 3:**
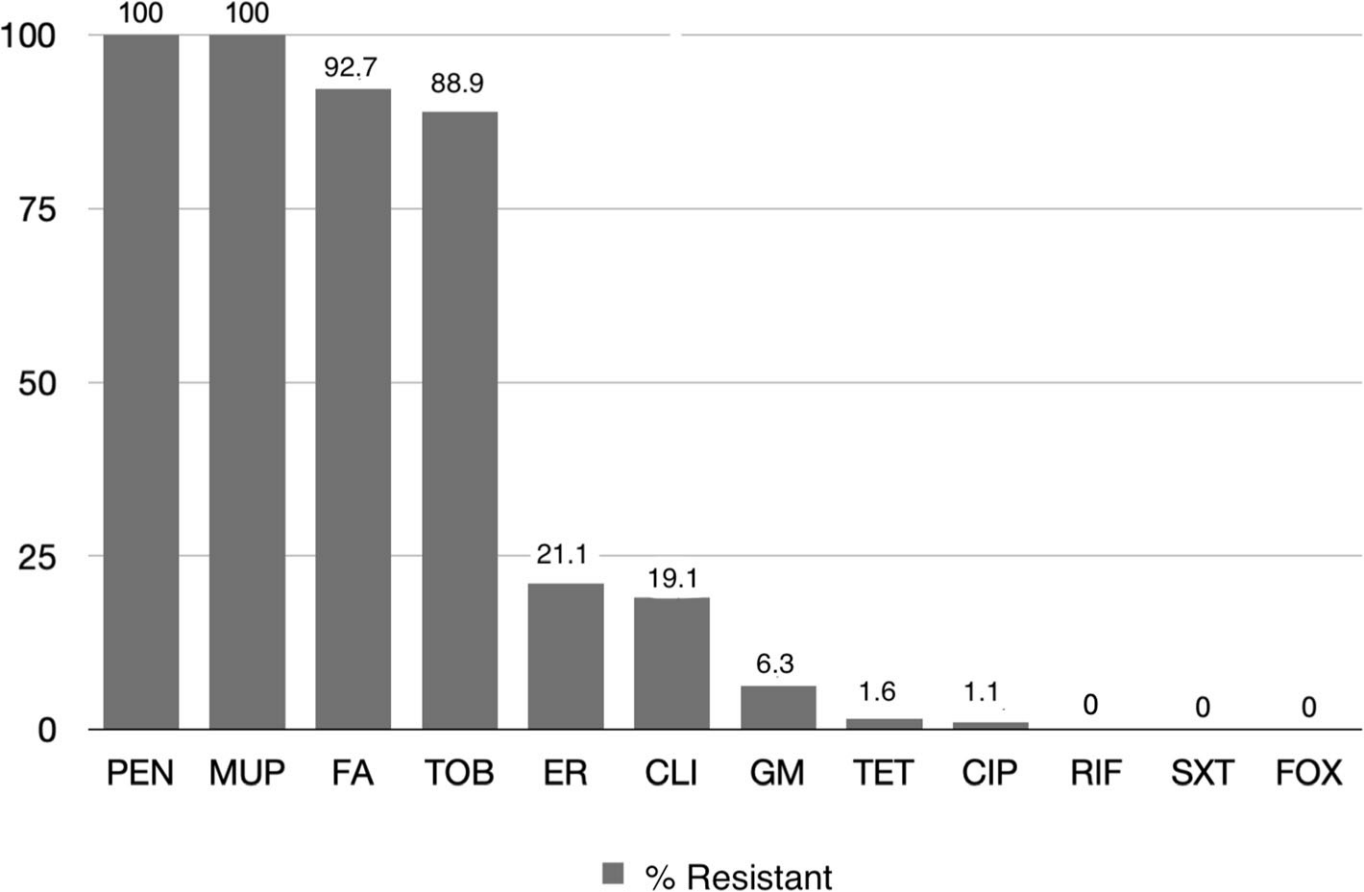
Antimicrobial resistance patterns of mupirocin-resistant MSSA from SSTIs. Antimicrobials used: penicillin (PEN), mupirocin (MUP), fusidic acid (FA), tobramycin (TOB), erythromycin (ER), clindamycin (CLI), gentamicin (GM), tetracycline (TET), ciprofloxacin (CIP), rifampicin (RIF), sulfamethoxazole/trimethoprim (SXT), cefoxitin (FOX)

One third of MSSA, 1484/4806 (30.88 %), associated with SSTIs showed mupirocin MICs ranging from 64 mg/L to > 1024 mg/L. Most isolates (1423/1484, 95.9 %) exhibited high-level resistance (> 512 mg/L) to mupirocin. From 2014 to 2017, an increase of mupirocin-resistant isolates among MSSA causing SSTIs was observed, from 16.45 to 38.66 %, followed by a decrease in 2018 (33.7 %) (Fig. 2). All mupirocin-resistant isolates were also penicillin-resistant, whereas, 1376/1484 were also resistant to fusidic acid (92.7 %), and 1320 to tobramycin (88.9 %) (Fig. 3). Three hundred and thirteen expressed resistance to erythromycin (21.1 %), out of which 283 were also clindamycin-resistant (184 exhibited constitutive and 99 inducible resistance). The majority of isolates was multi-resistant; however, all were susceptible to rifampicin and sulfamethoxazole/trimethoprim (Fig. 3).

From the total of 260 molecularly analyzed isolates, 181 were recovered from children and 79 from adults; all were *mupA*-positive, while all fusidic acid-resistant isolates (241/260) carried *fusB* and the tobramycin-resistant ones (230/260) carried *ant(4′)-Ia*. From the 55/260 erythromycin-resistant isolates, 50 were also resistant to clindamycin; 51/55 carried *ermC* and four the *ermA* gene. The five erythromycin-resistant/clindamycin-susceptible strains carried *mrsA* gene encoding the MsrA efflux pump.

**Fig. 4:**
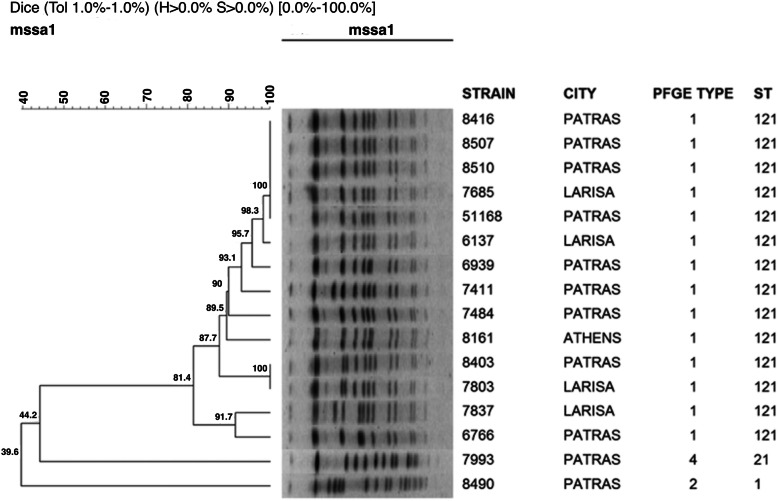
Dendrogram of representative mupirocin-resistant MSSA strains

PFGE typing revealed a mostly unvarying population; the 260 *S. aureus* were grouped into three PFGE types. One major pulsotype (type 1) was identified comprising of 258/260 (99.2 %) tested strains. MLST revealed that this pulsotype was classified to sequence type ST121. One isolate recovered from a patient with skin lesions was classified as PFGE type 2, ST1. Another *S. aureus* strain, also from skin lesions, belonged to PFGE type 4, ST21 (Fig. 4). ST1 is the primary group founder of clonal complex 1 and ST21 belongs to clonal complex 22.

The majority of the 260 tested isolates carried *eta* (244, 93.85 %), *etb* (255, 98.08 %) and 231 (88.85 %) also carried *fnbA*. From the 258 isolates which belonged to ST121, 242/258 (93.8 %) carried both *eta* and *etb*, two (0.78 %) carried only *eta*, whereas, another 13/258 (5.04 %) were only *etb*-positive; nine among them carried the PVL-encoding genes, all isolated in PAKCH during 2014–2016. One ST121 strain was negative for exfoliative and PVL toxin genes. Most ST121 *S. aureus* (88.76 %) carried also *fnbA*. The unique strain classified as ST1 was PVL-and *fnbA*-positive but negative for the exfoliative toxin genes, while the ST21 strain was negative for all the toxin genes tested. In Table 2 the molecularly analyzed isolates are presented according to patients’ age and clinical entities.

**Table 2:** Clonal and toxin distribution of the molecularly analyzed strains in relation to patients’ age and clinical entities

	Clinical entities	Clones	*PVL*	*eta*	*etb*	*fnbA*
**Children (181)**	Nonbullous impetigo (55)	ST121 (55)	-	54	53	55
	Bullous impetigo (35)	ST121 (35)	-	34	35	33
	Nasal (28)	ST121 (28)	-	26	28	27
	SSSS (13)	ST121 (13)	-	13	13	12
	Paronychia (11)	ST121 (11)	-	9	11	9
	Furunculosis (8)	ST121 (8)	-	6	8	8
	Abscess (8)	ST121 (8)	7	7	8	6
	Infected dermatitis (7)	ST121 (7)	-	6	7	6
	Otorrhea (5)	ST121 (5)	-	5	5	4
	Ophthalmia (4)	ST121 (4)	-	4	4	3
	Vaginitis (3)	ST121 (3)	-	-	2	2
	Omphalitis (3)	ST121 (3)	-	3	3	2
	Cellulitis (1)	ST121 (1)	-	1	1	1
**Adults (79)**	Infected dermatitis (27)	ST121 (26)	-	26	26	23
		ST21 (1)	-	-	-	-
	Paronychia (27)	ST121 (27)	-	26	27	20
	Furunculosis (20)	ST121 (20)	1	20	20	17
	Abscess (2)	ST121 (1)	1	1	1	-
		ST1 (1)	1	-	-	1
	Nasal (2)	ST121 (2)	-	2	2	2
	Mastitis (1)	ST121 (1)	-	1	1	-
**Total (260)**			**10**	**244**	**255**	**231**

## Discussion

In this study, *S. aureus* recovered from patients with SSTIs among three tertiary-care hospitals in Greece were analyzed in terms of antimicrobial resistance and genetic diversity. *S. aureus* was the main etiologic agent of SSTIs during the five-year period, isolated from 46.4 % of the cases, whereas, the majority was MSSA (78.2 %). Studies worldwide have demonstrated the virulence of *S. aureus* and its association with SSTIs [1, 2, 5, 8–10, 14]. Determination of clonal distribution and virulence factors of community-acquired *S. aureus* clinical isolates from purulent SSTIs in Beijing, China showed MSSA prevalence (79.6 %), in agreement to our results [15].

The increasing resistance of staphylococci to antimicrobials is also well described. Approximately one-third of *S. aureus* from SSTIs and nasal colonization from patients within 10 primary care clinics in South Texas, USA, were multi-drug resistant, especially isolates causing SSTIs as compared to nasal colonizers (37 % versus 11 %, respectively) [16]. In our study, the majority of *S. aureus* even though MSSA, were multi-resistant, expressing resistance to penicillin, mupirocin, fusidic acid (92.7 %), tobramycin (88.9 %) and 21.1 % to macrolides.

The resistance of staphylococcal isolates to mupirocin in Greece was low in 2002 (2 %) [17] and it is increasing from 2010; a six-year (2010–2015) retrospective review of *S. aureus* skin infections in Athens’ hospitals showed that 11.4 % of MSSA were resistant to mupirocin [18]. In 2014, ST121 mupirocin-resistant clone has emerged among children attending PAKCH, in Athens [14]. In the present multi-centre study, mupirocin, fusidic acid, tobramycin and macrolide resistance was associated with *mupA*, *fusB, ant(4′)-Ia* and *erm* carriage, respectively. In our tertiary care centers, an annual increase of mupirocin-resistant MSSA recovered from patients with SSTIs was observed from 2014 to 2017, with a slight decrease in 2018. Overall, the resistance to mupirocin was 30.88 % among MSSA in SSTIs-associated isolates. Two mechanisms of high-level mupirocin resistance have been detected; the first one is mediated by acquisition of a plasmid-mediated *mupA* or *ileS2* gene, whereas, the second is due to *mupB* gene (showing 65 % similarity with *mupA*). Mupirocin resistance has been increasing among staphylococci in many parts of the world and it seems to be more common among MRSA due to prior mupirocin use [14, 19]. Resistance to fusidic acid due to the presence of *fusB* is highly indicative of ST121 clonal spread, as it was firstly detected in PAKCH [14].

Aminoglycosides’ resistance rate and AMEs gene presence in *S. aureus* may be due to the predominance of specific clones but also to aminoglycosides’ usage in each area combined with a possible horizontal AMEs gene transfer, as reported in a previous Greek study, where *aph(3′)-IIIa* predominated [20]. Therefore, the detection of tobramycin-resistant/gentamicin-susceptible isolates carrying *ant(4′)-Ia* gene in the present study, besides clonal spread, may also reflect aminoglycosides’ usage.

The prevalence of *ermC* has been previously reported in Greece among macrolide-resistant staphylococci, whereas, the rate of erythromycin resistance was statistically significantly lower in MSSA (20.7 %) than in MRSA (58.6 %) and similar to our findings (21.1 %) [20, 21].

Fibronectin-binding protein A (FnBPA) mediates adhesion of *S. aureus* to fibrinogen, elastin and fibronectin, favoring the establishment of staphylococcal infections. In a study from Turkey, *fnbA* was detected in 77.7 % of strains from wound specimens, a lower percentage as compared to our study (88.85 %) [22].

Exfoliative toxins are implicated in impetigo, a common superficial skin infection in children and rarely in the development of the staphylococcal-scalded skin syndrome (SSSS), also known as Ritter disease, a generalized blistering skin disorder. The condition is mostly observed in neonates and children younger than 5 years with a peak between 2 and 3 years of age [23]. The majority of our 260 molecularly tested isolates carried *eta* (93.85 %) and *etb* (98.08 %) but only 10/260 tested isolates were PVL-positive. In an epidemiological study among MRSA in Greece over a 12-year period (2001–2012), an increasing rate of MRSA among *S. aureus* infections was detected up to 2008, encoding mainly PVL (63.85 %) [2]. Similarly to our findings, in a collection of pediatric MSSA from SSTIs over a 43-month period in Athens, Greece, 93 % of isolates carried both *eta* and *etb* [14]. In a collection of *S. aureus* from SSTIs among pediatric patients referred to the Children’s Medical Center Hospital in Tehran, during one-year period (2017–2018), *eta* was the most prevalent gene with a high occurrence (100 % in both MRSA and MSSA) and *etb* was found in only 23.8 % of the MSSA population, whereas in our study *etb* was the predominant toxins’ gene in MSSA [24]. It is remarkable that almost all staphylococcal strains harbor both the *eta* and *etb* genes, in accordance with the study conducted in a single center (PAKCH) [14], whereas only 0.5 to 3 % of MSSA strains examined in previous studies carried those genes [25].

Regarding clonal dissemination in the present study, the vast majority of the molecularly analysed strains belonged to a single clone, ST121, which has been identified in several studies worldwide [14, 26–31]. The emergence of this predominant MSSA clone, resistant to mupirocin and highly resistant to tobramycin and fusidic acid is associated with a sharp increase in SSSS cases in Greece since 2015, with 31 cases firstly documented in a children’s hospital in Athens during 2014–2017 [14, 26]. Presumably, the indiscriminate and repetitive use of mupirocin as an over-the-counter agent, drives the selection of this clone, as shown in a previous work from pediatric mupirocin-resistant isolates where 32.3 % of patients reported past use of topical antimicrobials, with 20.6 % of lesions being recurrent [14].

ST121 was identified in several countries among *S. aureus* causing SSTIs, such as, Paraguay, New Caledonia, Togo, France, Czech Republic, Germany, Turkey, US, Fr. West Indies, UK, Polynesia, Switzerland, Spain, Algeria, The Netherlands and China showing an overall prevalence rate approximately 10 % in MSSA, and less than 5 % in MRSA [27, 28]. In Portugal, in a study among children with SSTIs, 63 % of *S. aureus* belonged to ST121 [29]. In Yangon, Myanmar, ST121 was found among pvl-positive MSSA, in contrast to our MSSA collection [30]. In agreement to our results, in Houston, Texas, *S. aureus* recovered from children with SSSS revealed that 40/58 isolates belonged to ST121, while 11 more strains belonged to STs from the same Clonal Complex (CC121), the majority being MSSA carrying *eta* and *etb* [31].

In the present collection of strains, only 10/260 *S. aureus* were PVL-positive. This is associated with the vast predominance of ST121 carrying mainly *eta*/*etb* genes, over other MSSA clones (ST1 and ST21). ST1 has been reported as one of the main clones among MSSA in a study from Guangzhou, China [32]. An investigation on the global population structure of PVL-positive MSSA from five continents up to 2007, revealed that the most frequent STs were ST30 (19.9 %) and ST121 (19.9 %) [33]. In our study only 2/260 of strains were classified in other sequence types; one strain carrying only PVL was identified as ST1 and another strain, negative for all the toxins’ genes tested, as ST21. In a previous Greek study, we found that 45 % of MRSA and only 12 % of MSSA carried the PVL genes [34]. Still, MSSA populations can be PVL-positive in majority, as reported by Darboe et al., among Gambian patients with invasive and non-invasive infections, where PVL-positive strains accounted for 61.4 % (180/293) of *S. aureus* isolates [35].

A limitation of the present study is that the great majority of patients were admitted at the Departments for outpatients and therefore no data were available about any therapeutic failures or alternative treatments.

## Conclusions

The dominant ST121 clone in our study, associated with high-level resistance to mupirocin, prevailed among mupirocin-resistant staphylococcal isolates from SSTIs. This successful clone, comprised of strains carrying resistance determinants for topical antimicrobials, exfoliative toxins and adhesin genes, affects adult and pediatric patients in three different areas of Greece during the five-year period. Clinicians should be aware of this clone whereas, there is need for de-escalation of mupirocin use in the community.

## Methods

### Patients and hospitals

A total of 6145 *S. aureus* were recovered during a five-year period (2014–2018) from 13,244 patients with SSTIs admitted in three tertiary-care hospitals in different areas of Greece, serving about one third of the population; the University General Hospital of Patras (UGHP: 2579 isolates, 770 beds, with approximately 37,000 admissions per year in Southwestern Greece), P. & A. Kyriakou Children’s Hospital in Athens that is a tertiary care referral center for children (PAKCH: 1964 isolates, 400 beds, 16,000 admissions) and the University General Hospital of Larissa (UGHL: 1602 isolates, 650 beds, 35,000 admissions, in Central Greece). Children’s hospitals admit patients up to the age of 16 years.

Skin infections were classified as secondarily infected dermatitis, impetigo (bullous and nonbullous), furunculosis or scalded skin syndrome. Soft tissue infections such as cellulitis and abscess, otorrhea, ophthalmia, vaginitis, omphalitis, mastitis, and paronychia were also included in the study. Staphylococcal strains isolated from nasal cultures were included in the analysis only if skin lesions were clinically relevant (e.g., multiple skin lesions). The Ethics Committees of the UGHP, PAKCH and UGHL approved this study and waived the need for informed consent (Approval Numbers: 786, 9956 and 1355, respectively).

### Phenotypic identification and antibiotic susceptibility testing

Staphylococci were identified to species level based on colony morphology, Gram staining, catalase production, coagulase testing (Slidex Staph Plus; bioMérieux, Marcy l’ Etoile, France) and by the Vitek2 System (GP card, bioMerieux). Susceptibility to cefoxitin (FOX), penicillin (PEN), erythromycin (ER), clindamycin (CLI), tobramycin (TOB), gentamicin (GM), ciprofloxacin (CIP), fusidic acid (FA), rifampicin (RIF), tetracycline (TET) and sulfamethoxazole/ trimethoprim (SXT), was tested by the disk diffusion method according to EUCAST guidelines [36]. Testing for inducible and constitutive lincosamide resistance was performed with the D-test. MICs of mupirocin (MUP) and oxacillin (OX) were determined by E-test® (bioMerieux). Isolates resistant to at least three classes of antimicrobials were considered multidrug resistant. Phenotypic determination of MSSA was based on cefoxitin disk susceptibility [36]. Isolates with mupirocin MIC > 1 mg/L were further analysed.

### Molecular analysis

Two hundred and sixty representative mupirocin-resistant isolates were sent to the National Reference Laboratory for Staphylococci for molecular analysis; 110 isolates from the UGHP, 47 isolates from the UGHL and 103 isolates from the PAKCH. Strains were selected according to the sample size of participating hospitals, age and gender of patients, clinical specimen and date of sampling, infection site, clinical entities, hospital ward, and whether the isolate was epidemiologically representative of a cluster, including colonizing ones.

Amplification of genes encoding Panton-Valentine Leukocidin (PVL, *lukS*/*lukF-*PV), exfoliative toxins (*eta*, *etb*), adhesin FnbA (*fnbA*) and the resistance genes *mupA* (encoding high-level resistance to mupirocin), *fusB* (fusidic acid), *ermA, ermC* and *msrA* (macrolides/lincosamides), as well as *aac(6′)-Ie-aph(2′′)*, *ant(4′)-Ia* and *aph (3′)-IIIa* encoding aminoglycosides’ modifying enzymes (AMEs), was performed by PCRs with specific primers, as described. PCR products were analyzed by electrophoresis on 1 % agarose gels. *S. aureus* strains ATCC 49,775 (PVL-positive), Fri913 (*fnbA-*positive), A920211 (*eta/etb-*positive, *mupA-*positive), were used as controls for the PCRs [20, 21, 37–40].

### Clonal identification

From the 260 representative strains, DNA extraction was performed into agarose disks and strains were classified into pulsotypes by pulsed-field gel electrophoresis (PFGE) of chromosomal DNA *SmaI* digests performed in a CHEF DR III apparatus (Bio-Rad, Richmond, CA), as previously described [41]. A dendrogram comparing molecular weights of strains’ DNA fragments was performed by FPQuest software version 4.5 (Bio-Rad Laboratories Inc). According to criteria established by Miragaia et al. [42] patterns differing by less than 79 % (corresponding to a difference of less than seven bands) were considered to belong to the same PFGE type.

The selected *S. aureus* strains were also characterized by Multilocus Sequence Typing (MLST) (http://mlst.net) [43]. The results were analyzed by the application of eBURST algorithm. Clonal complexes were defined by using the default setting, in which all STs within a clonal complex differ by no more than one allele from at least one other ST in the clonal complex.

### Statistical analysis

Counts and percentages are reported for categorical variables. Statistical analysis was performed using SPSS for Windows (version 18; SPSS, Chicago, IL).

## Supplementary Information

**Additional file 1: Supplementary Figure 1:** PFGE of *S. aureus* after DNA digestion with *Sma*I.

## Data Availability

The datasets used and/or analyzed during the current study are available from the corresponding author on reasonable request.

## Abbreviations

AMEs: Aminoglycosides’ modifying enzymes
CIP: Ciprofloxacin
CLI: Clindamycin
ER: Erythromycin
EUCAST: European Committee on Antimicrobial Susceptibility Testing
FA: Fusidic acid
FnBPA: Fibronectin-binding protein A
FOX: Cefoxitin
GM: Gentamicin
MIC: Minimum inhibitory concentration
MLST: Multilocus Sequence Typing
MSCRAMMs: Microbial Surface Components Recognizing Adhesive Matrix Molecules
MRSA: Methicillin-resistant *Staphylococcus aureus*
MSSA: Methicillin-susceptible *Staphylococcus aureu*s
MUP: Mupirocin
OX: Oxacillin
PAKCH: P. & A. Kyriakou Children’s Hospital
PCR: Polymerase chain reaction
PEN: Penicillin
PFGE: Pulsed-field gel electrophoresis
PVL: Panton-Valentine leukocidin
RIF: Rifampicin
SPSS: Superior Performance Software System
SSSS: Staphylococcal scalded skin syndrome
SSTIs: Skin and soft tissue infections
ST: Sequence type
SXT: Sulfamethoxazole/ trimethoprim
TET: Tetracycline
TOB: Tobramycin
UGHL: University General Hospital of Larissa
UGHP: University General Hospital of Patras
