# Supplementary material for: Emergence of a mupirocin-resistant, methicillin-susceptible Staphylococcus aureus clone associated with skin and soft tissue infections in Greece
Source: BMC Microbiol. 2021 Jul 3;21:203. doi: 10.1186/s12866-021-02272-5 (PMC8254358; doi:10.1186/s12866-021-02272-5)
Supplement: Supplementary file 1 — Additional file 1: Supplementary Figure 1: PFGE of S. aureus after DNA digestion with SmaI. [file 12866_2021_2272_MOESM1_ESM.pptx]

## Slide 1
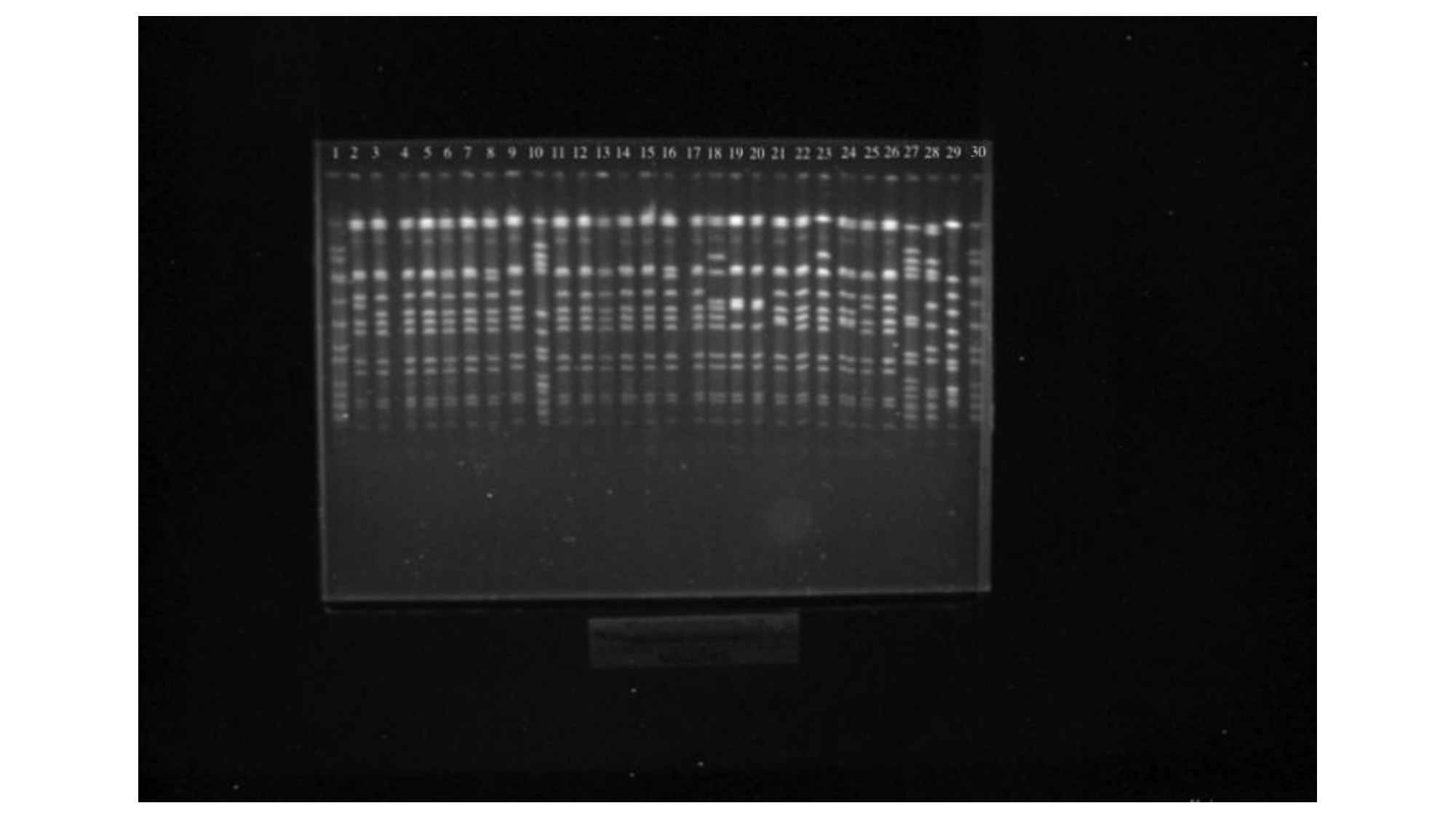

## Slide 2
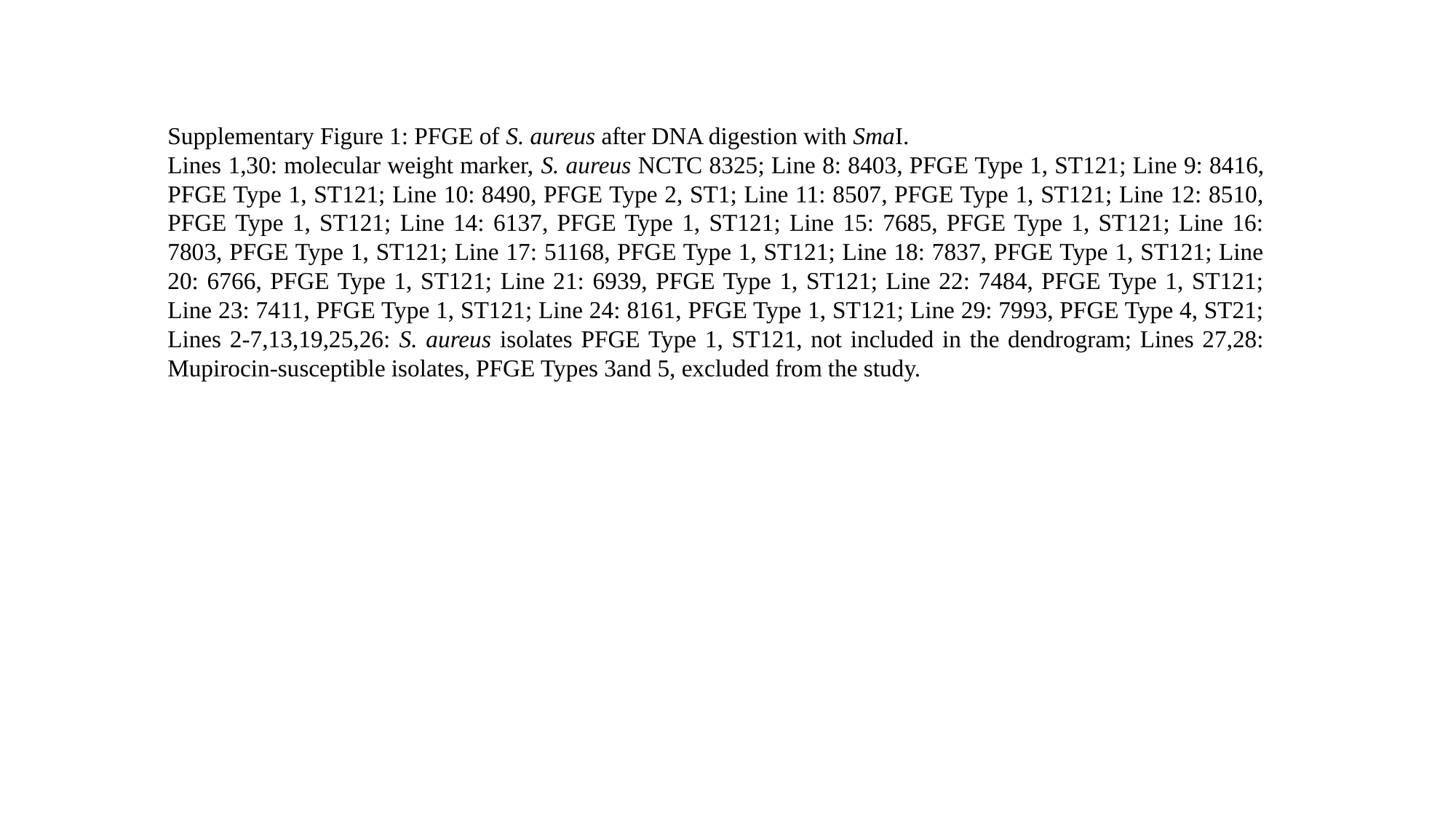

Supplementary Figure 1: PFGE of S. aureus after DNA digestion with SmaI.
Lines 1,30: molecular weight marker, S. aureus NCTC 8325; Line 8: 8403, PFGE Type 1, ST121; Line 9: 8416, PFGE Type 1, ST121; Line 10: 8490, PFGE Type 2, ST1; Line 11: 8507, PFGE Type 1, ST121; Line 12: 8510, PFGE Type 1, ST121; Line 14: 6137, PFGE Type 1, ST121; Line 15: 7685, PFGE Type 1, ST121; Line 16: 7803, PFGE Type 1, ST121; Line 17: 51168, PFGE Type 1, ST121; Line 18: 7837, PFGE Type 1, ST121; Line 20: 6766, PFGE Type 1, ST121; Line 21: 6939, PFGE Type 1, ST121; Line 22: 7484, PFGE Type 1, ST121; Line 23: 7411, PFGE Type 1, ST121; Line 24: 8161, PFGE Type 1, ST121; Line 29: 7993, PFGE Type 4, ST21; Lines 2-7,13,19,25,26: S. aureus isolates PFGE Type 1, ST121, not included in the dendrogram; Lines 27,28: Mupirocin-susceptible isolates, PFGE Types 3and 5, excluded from the study.
